# Close of the Volume

**Published:** 1858-08

**Authors:** 


### EDITORIAL AND MISCELLANEOUS.

### Close of the Volume.

With this number will close the third volume of our Journal, and we would, in this connection, suggest to our friends, that this is a most appropriate time for effort in extending our subscription list. Each subscriber could, doubtless, *add one* most readily, and if accompanied with the cash, it would, at once, enable us to furnish a Journal of much greater merit.

We know that a large number of our subscribers feel a special interest in the prosperity of the Journal, and need only to be reminded of the importance of their efforts to induce them to aid in its circulation. We shall then hope before the next number (which commences the 4th vol.) is issued, to receive a large addition to our subscription list.

---

☞ We have a number of books on hand, sent for review, which it was our intention to have noticed in this number of our Journal, but have been compelled to defer it, until we shall be able to give them the attention they merit.

